# One dose of Psilocybin modulates Brain Activity among Depressive patients : Preliminary Neuroimaging Results from the PSILODAC Study

**DOI:** 10.1192/j.eurpsy.2026.10729

**Published:** 2026-07-31

**Authors:** M. Pastre, F. Pereira, T. Gibert, A. Giron, B. Goemare, E. Olié, P. Courtet, J. Lopez-Castroman, I. Conejero

**Affiliations:** 1 Psychiatry, CHU Nimes, Nimes; 2 Institute of Functional Genomics; 3 Psychiatry, CHU Montpellier, Montpellier, France; 4 University of Santiago de Compostela, Santiago de Compostela, Spain

## Abstract

**Introduction:**

Treatment-resistant depression (TRD) remains a major clinical challenge, with limited efficacy of available therapies. Psilocybin, a serotonergic psychedelic, has shown promising antidepressant effects. As recent studies have shown altered default mode network activity following psilocybin administration, further neuroimaging research in clinical populations is needed to clarify its mechanisms of action and the neural networks associated with symptom improvement in depression.

**Objectives:**

To investigate whether the antidepressant effects of psilocybin are associated with changes in resting-state brain activity and to assess the feasibility of conducting psilocybin trials in a French clinical population.

**Methods:**

Patients meeting DSM-IV criteria for moderate to severe TRD underwent two fMRI sessions 2 days before and 5 days after a single oral psilocybin administration (25 mg). Amplitude of Low Frequency Fluctuations (ALFF) and regional homogeneity analyses were conducted to identify changes in resting brain activity after psilocybin.

**Results:**

Ten patients underwent the two fMRI sessions (8 women, 1 man; mean age 42.2 years). Resting-state analyses revealed increased ALFF signal in the right Ventro-Lateral Pre-Frontal Cortex (vlPFC) (Figure 1), the right Medial Pre-Frontal Cortex (mPFC) (Figure 2), and the left Inferior Parietal Lobule (IPL) (Figure 3) after psilocybin administration. Regional homogeneity analyses confirmed greater signal coherence in overlapping regions.

**Image 1:**
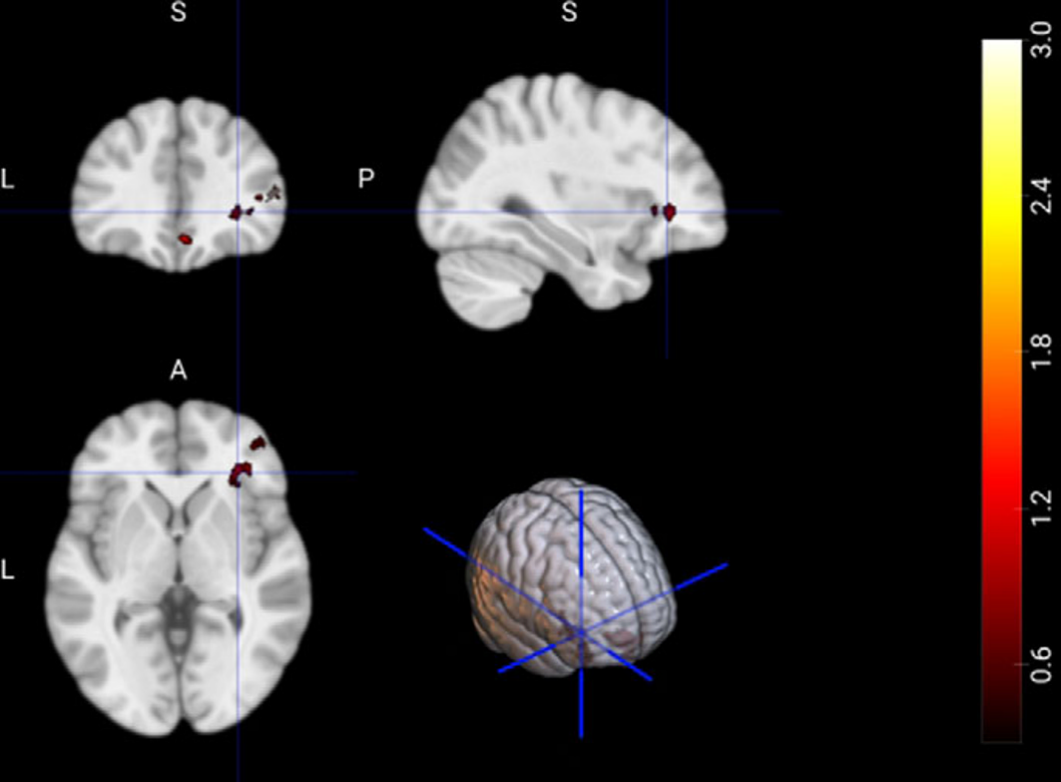
Image 1: Long description.

**Image 2:**
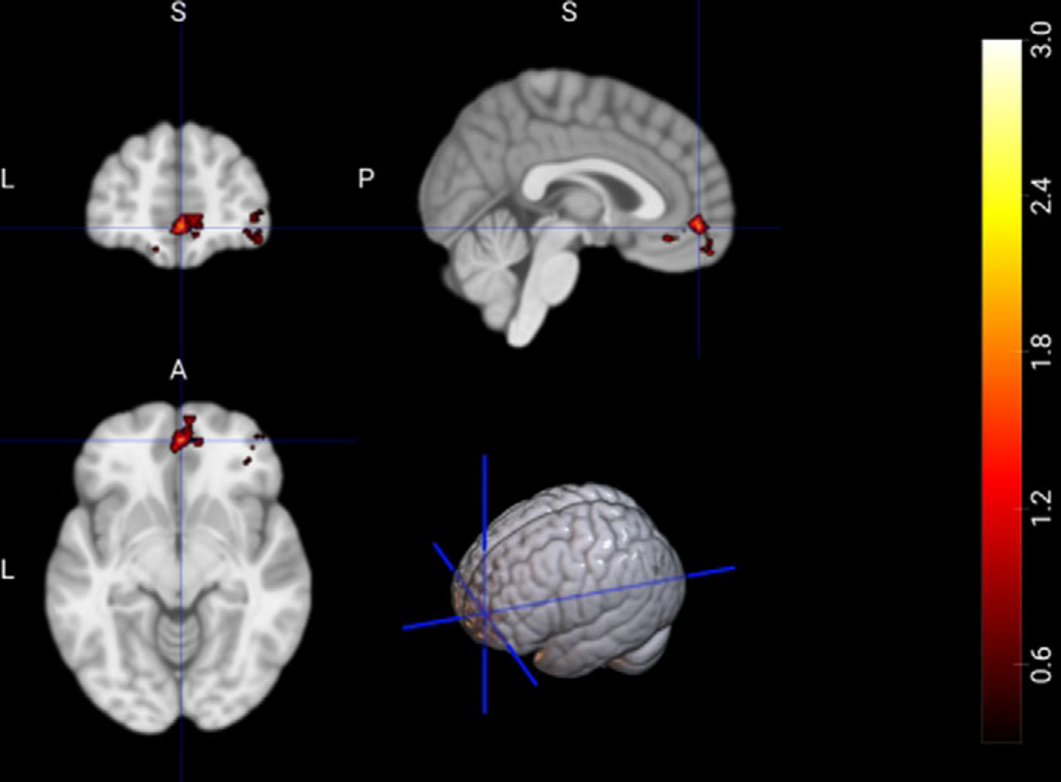
Image 2: Long description.

**Image 3:**
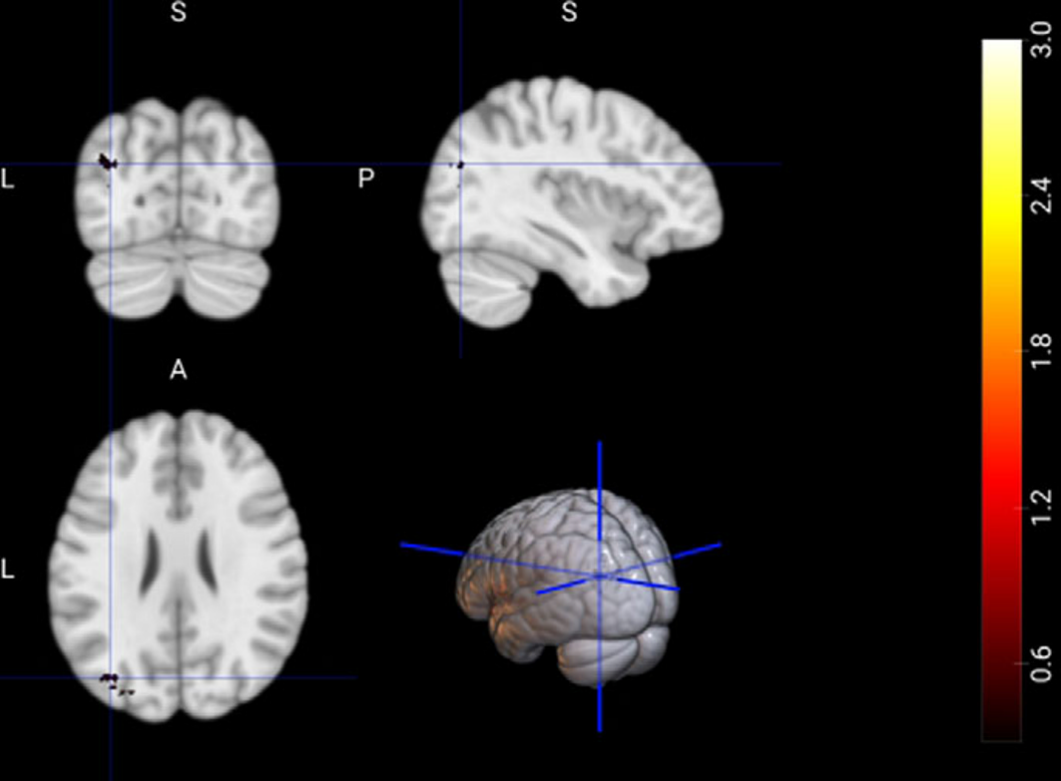
Image 3: Long description.

**Conclusions:**

Preliminary results from the PSILODAC study suggest that psilocybin modulates resting-state brain activity in patients with TRD. Upcoming analyses will include resting-state functional connectivity within positive valence circuits, as well as correlations between imagery outcomes and clinical response to psilocybin.

**Disclosure of Interest:**

None Declared

## Long descriptions

### Image 1: Long description

A multi-panel M R I visualization showing neural activation.

* Top-left panel: Coronal cross-section labeled S at the top and L on the left. Blue crosshairs intersect at a small red activation cluster in the right hemisphere, with a second smaller cluster located inferiorly near the midline.

* Top-right panel: Sagittal cross-section labeled S at the top and P on the left. The blue vertical crosshair identifies a red activation cluster in the frontal region.

* Bottom-left panel: Axial cross-section labeled A at the top and L on the left. The blue crosshairs pinpoint a cluster of red and yellow activation in the right frontal cortex.

* Bottom-right panel: A 3 D rendered model of the brain with blue coordinate axes intersecting at the site of interest on the cortical surface.

* Right-side scale: A vertical color gradient bar ranging from dark red at the bottom (0.6) to bright white-yellow at the top (3.0), representing the intensity of the signal.

Navigate back to Image 1:.

### Image 2: Long description

The figure consists of four panels showing an M R I brain scan with blue crosshair markers and red-to-yellow heat map clusters indicating activation levels.

* Top-left panel: A coronal view labeled with S for Superior and L for Left. A significant cluster of activation is centered at the intersection of the blue crosshairs in the subcortical region, with smaller clusters to the right.

* Top-right panel: A sagittal view labeled with S for Superior and P for Posterior. The crosshairs intersect at a cluster in the frontal lobe, near the anterior surface.

* Bottom-left panel: An axial view labeled with A for Anterior and L for Left. The crosshairs pinpoint a large activation cluster in the medial frontal region.

* Bottom-right panel: A three-dimensional surface rendering of the brain with blue axes intersecting at the frontal pole, showing the external location of the activation.

* Far-right: A vertical color bar scale ranging from 0.6 at the bottom in dark red to 3.0 at the top in bright white-yellow. The scale has numerical markers at 0.6, 1.2, 1.8, 2.4, and 3.0.

Navigate back to Image 2:.

### Image 3: Long description

A multi-panel M R I scan displays brain activity across four views.

* Top-left panel (Coronal view): Labeled S at the top and L on the left. A blue crosshair intersects in the upper-left quadrant of the brain slice, highlighting a small dark cluster of activation.

* Top-right panel (Sagittal view): Labeled S at the top and P on the left. The blue crosshair is positioned near the posterior edge of the cortex, marking the same activation site from a side profile.

* Bottom-left panel (Axial view): Labeled A at the top and L on the left. The crosshair is located in the lower-left region (posterior-lateral), showing the cluster near the outer edge of the brain tissue.

* Bottom-right panel (3 D Render): A three-dimensional model of the brain with blue axes intersecting at the specific anatomical location of the activation.

On the far right, a vertical color scale bar ranges from 0.6 at the bottom to 3.0 at the top. The gradient transitions from dark red at the bottom, through bright orange and yellow, to white at the top, representing the intensity of the signal.

Navigate back to Image 3:.

